# A time-driven activity-based costing approach for identifying variability in costs of childbirth between and within types of delivery

**DOI:** 10.1186/s12884-021-04134-4

**Published:** 2021-10-20

**Authors:** Kathia Dubron, Mathilde Verschaeve, Filip Roodhooft

**Affiliations:** 1 KU Leuven, University Hospital Leuven, Kapucijnenvoer 33, 3000 Leuven, Belgium; 2 KU Leuven, Faculty of Economics and Business, Research Centre Accountancy, Leuven, Belgium; 3 Vlerick Business School, Accounting and Finance, Gent, Belgium

**Keywords:** Obstetrics, Time-driven activity-based costing, Vaginal birth, Caesarean section, Health management

## Abstract

**Background:**

Recently, time-driven activity-based costing (TDABC) is put forward as an alternative, more accurate costing method to calculate the cost of a medical treatment because it allows the assignment of costs directly to patients. The objective of this paper is the application of a time-driven activity-based method in order to estimate the cost of childbirth at a maternal department. Moreover, this study shows how this costing method can be used to outline how childbirth costs vary according to considered patient and disease characteristics. Through the use of process mapping, TDABC allows to exactly identify which activities and corresponding resources are impacted by these characteristics, leading to a more detailed understanding of childbirth cost.

**Methods:**

A prospective cohort study design is performed in a maternity department. Process maps were developed for two types of childbirth, vaginal delivery (VD) and caesarean section (CS). Costs were obtained from the financial department and capacity cost rates were calculated accordingly.

**Results:**

Overall, the cost of childbirth equals €1894,12 and is mainly driven by personnel costs (89,0%). Monitoring after birth is the most expensive activity on the pathway, costing €1149,70. Significant cost variations between type of delivery were found, with VD costing €1808,66 compared to €2463,98 for a CS. Prolonged clinical visit (+ 33,3 min) and monitoring (+ 775,2 min) in CS were the main contributors to this cost difference. Within each delivery type, age, parity, number of gestation weeks and education attainment were found to drive cost variations. In particular, for VD an age >  25 years, nulliparous, gestation weeks > 40 weeks and higher education attainment were associated with higher costs. Similar results were found within CS for age, parity and number of gestation weeks.

**Conclusions:**

TDABC is a valuable approach to measure and understand the variability in costs of childbirth and its associated drivers over the full care cycle. Accordingly, these findings can inform health care providers, managers and regulators on process improvements and cost containment initiatives.

**Supplementary Information:**

The online version contains supplementary material available at 10.1186/s12884-021-04134-4.

## Background

Childbirth is one of the main causes of hospital admission for women, representing about 5% of hospital expenditures in OECD countries [1, 2]. Meanwhile, prior studies have reported wide variability in the provision of maternal care, leading to fluctuations in overall childbirth costs [3–5]. This phenomenon has generated substantial interest, with researchers particularly focusing on facility-related characteristics and their impact on cost variability [6–8]. However, the available literature offers less information about the factors driving cost variation within hospitals. Given the overall pressures on health care expenditures, it is important to have a more nuanced understanding of the different costs incurred over the complete treatment path of childbirth, as well as factors driving cost variations in order to guide effective pricing and process improvement decisions.

The objective of this study is therefore twofold. First, this study describes the application of a time-driven activity-based costing model to estimate the costs of childbirth over the full cycle of care at the maternity department from the viewpoint of the hospital. The second goal of this paper is to outline how the TDABC costing method can provide insights into how childbirth costs vary according to considered disease and patient characteristics. Recently, time-driven activity-based costing was suggested as an alternative, more accurate method to calculate hospital costs as it allows the assignment of costs directly to patients [9, 10]. The method is relatively easy to implement since it only requires two parameters: (1) cost per time unit of supplying resources to activities and (2) the time required by the resource to carry out one unit of activity. By the use of multiple time drivers, TDABC enables the design of cost models that capture the complexity of processes far better than traditional accounting methods. In particular, process maps allow the incorporation of variation caused by underlying determinants along the processes. Hence, it is a well-suited costing method to track the expenses involved in the intricacy of activities in organizations such as hospitals [9, 10].

This study focuses on two types of birth, vaginal delivery (VD) and caesarean section (CS), which are labelled as ‘disease’ characteristics in the remainder of this paper. Based on medical literature review, four patient characteristics that were found to influence the clinical pathway are included in the analysis. In particular, age, parity, gestation weeks and education attainment were put forward as they were associated with various obstetric complications and additional patient care [6, 11–20].

## Methods

### Study design, setting and data collection

A prospective cohort study was performed at a maternity department located in Belgium. Pregnant women were followed from moment of admission for childbirth to discharge after labour in March and April 2019. To estimate the cost of childbirth, a time-driven activity-based costing approach was applied using data from multiple sources. Information on the clinical pathway along with the associated activities and utilized resources was prospectively collected through structured interviews with medical staff, including nurses, midwives and physicians [21]. Time estimates were obtained from direct observations of staff and are based on the average time spent per activity. All relevant cost data were retrieved from the financial department of the hospital. Patient characteristics were collected by means of a survey conducted by the midwives during intake of the patient (Supplementary files 1 and 2). All patients in the sample were treated by the same nursing team in one particular hospital, hence controlling for hospital and staff variance. Ethical approval from the Medical Ethical Review Committee of the hospital were acquired prior to any data collection. The research was conducted in compliance with the principles of the Declaration of Helsinki. This research adheres to the STROBE guidelines for observational studies, with the STROBE checklist reported in Supplementary file 3.

### Patient and disease characteristics

For disease characteristics, a distinction is made between the type of childbirth, being VD and CS. Patient characteristics included in this analysis are age, parity, weeks of gestation and education attainment. Age is divided into two groups, ≤ 25 years and >  25 years, based on maternal perinatal outcomes after 25 years and following conventions in previous studies on cost calculation [11, 12, 17]. Accordingly, a distinction is made between nulliparous and multiparous women. A woman falls within the category “long” when she delivers her baby after more than 40 gestation weeks, and “normal” otherwise [11–13, 19]. Lastly, a classification is made between high and low education attainment, with women having a university or college degree grouped as ‘high’.

### Costing analysis

This study follows the seven-step approach of Kaplan and Porter [9] for the estimation of childbirth cost over its full cycle of care. This roadmap is an adapted version of the TDABC model of Kaplan and Anderson in that it is specifically designed for TDABC applications in health care settings [22].

## Results

The sample of our study comprises twenty-three patients. None of the patients were smokers or had comorbidities such as COPD, asthma, diabetes, intoxications or pre-eclampsia. The descriptive statistics of the patient and disease characteristics of the studied patients are displayed in Table 1. Total expenses of the maternity department were €4,538,469,19 in the last year and the number of births was 1223 with 6% births by CS.

**Table 1:** Overview of patient and disease characteristics of the sample of women who gave birth

	Vaginal delivery (***n*** = 20)	Caesarean section (***n*** = 3)
**Age**		
≤ 25 years	7	2
> 25 years	13	1
**Parity**		
Nulliparous	7	2
Multiparous	13	1
**Weeks pregnancy**		
≤ 37 weeks	0	0
38–40 weeks	11	1
> 40 weeks	9	2
**Education level**		
High school	4	1
College	3	1
University	1	0
Unknown	13	1

Note: None of the patients were smokers, had COPD, asthma, diabetes, intoxications or pre-eclampsia

### Step 1: Select the medical condition

The medical condition of interest in this study is childbirth. In particular, the cost of childbirth for a woman in labour is estimated from the moment she is admitted to the hospital for childbirth until discharge after delivering the child. Pre-natal counselling and subsequent follow-up sessions are beyond the scope of this research.

### Step 2: Care delivery value chain

The care delivery value chain (CDVC) depicts the principal activities during childbirth and their respective locations. All women in labour follow a general clinical pathway in the process of childbirth, including pre-delivery, delivery and post-delivery phases. The principal activities and locations of the CDVC are further enclosed in the process map.

### Step 3: Process maps for each activity in patient care delivery

**Fig. 1:**
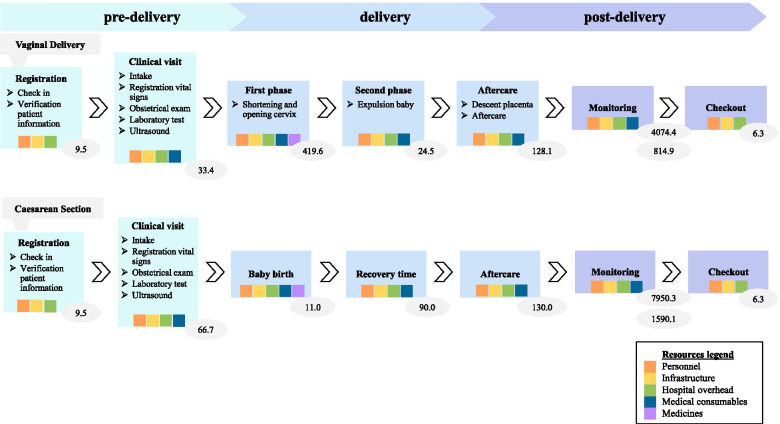
Process map for vaginal delivery and caesarean section. Legend: Large boxes show activities and color-coded squares show utilized resources per activity. Average time in minutes per activity is displayed in the ovals. For monitoring, lower and upper oval represent the total stay time after birth, and the nursing time by personnel, respectively

Two distinct process maps were developed reflecting the areas of variation between the two disease characteristics studied. Figure 1 presents the process maps of the cycle of care of VD and CS. For a CS, the activities, utilized resources and location in the pre-delivery and post-delivery phase are similar to the clinical pathway of VD. The pathway of the delivery phase, however, diverges. Rather than the first and second phase of the delivery, baby birth through surgical incision followed up by an additional recovery moment for the woman take place.

### Step 4: Time estimates for each process

The time estimates of each activity are included in the process maps in Fig. 1. For monitoring, two different time estimates are displayed, referring to the total time a patient stays in the hospital for monitoring after birth, and the time she is effectively being cared for by personnel during this stay, respectively. The duration of the activities along the pathway varies depending on disease characteristics. Time estimates of registration and check-out are equal between VD and CS. Clinical visit and monitoring after birth are estimated to consume more time for CS in comparison to VD. Baby delivery, in contrast, takes less time in CS. Within the two types of childbirth, variability in time estimates for patient characteristics is also observed but here not included in the process map. Further specification is provided in step seven.

### Step 5: Estimate the cost of supplying the resources

The maternity department identifies five cost categories: personnel, infrastructure, medical consumables, medicines and hospital overhead costs. For the calculation of childbirth cost, a division is made between direct and indirect costs. Direct costs are medical consumables and medicines and are directly allocated to the patient based on unit cost and average consumption. Indirect costs include personnel, infrastructure and hospital overhead costs. Personnel costs represent salaries of nurses, midwives and heads of the department. The costs of physicians are not considered in the scope of this study as physicians are not remunerated by the hospital itself but by the national health insurance fund and co-payment by patients. Infrastructure costs cover maintenance and depreciation costs of facilities and (large) medical equipment and machines. Hospital overhead costs consist of general costs such as transportation, cleaning supplies, photocopies, linen, and other (small) equipment. Allocation of indirect costs is based on how the activities utilize the resources, through multiplying its capacity cost rate (CCR) by the time spent by the resource.

### Step 6: Estimation of the capacity of each resource and calculate the capacity cost rate

The capacity cost rate represents the per minute cost of a resource and is calculated by dividing the total cost of the resource by its practical capacity. The practical capacity is the total amount of time a resource can actually be deployed for taking care of patients and in contrast to theoretical capacity, it accounts for unused time such as breaks, meetings, holidays, sick leave and machine-breakdowns. To obtain the capacity cost rate of each personnel category, the yearly salary cost is divided by the corresponding practical capacity per year. To estimate the capacity cost rate of infrastructure, the yearly infrastructure cost is divided by the product of the average number of child deliveries per year (1223) with the average length of stay of a patient entering for childbirth (4910 min). Similarly, the capacity cost rate of overhead hospital costs is estimated by the division of the yearly overhead costs by the same product of the average number of child deliveries per year with the average length of stay of a patient entering for childbirth. Table 2 summarizes the estimation of capacity cost rates for midwives, nursing staff, head of department, infrastructure and overhead hospital.

**Table 2:** Estimation of capacity cost rates for every cost category

	Yearly cost (€)	Practical capacity (min)	Capacity cost rate (€/min)
**Midwife**	75,712.71	219 days × 5.1 h = 67,014 min	1.13
**Nursing staff**	72,063.04	219 days × 5.1 h = 67,014 min	1.08
**Head of Department**	97,300.58	214 days × 5.6 h = 71,904 min	1.35
**Infrastructure**	90,864.99	1223 deliveries × 4910 min/delivery = 6,004,930 min	0.015
**Overhead hospital**	54,917.94	1223 deliveries × 4910 min/delivery = 6,004,930 min	0.009

Note: The average stay at the department of a patient entering for childbirth is calculated based on the yearly proportion of vaginal deliveries (96%) and caesarean sections (4%) in the studied hospital

### Step 7: Calculation of the total cost of patient care

The cost of childbirth for one patient is the total of all medicines, medical consumables, personnel, infrastructure, and hospital overhead costs consumed as the patient moves along the clinical pathway. A detailed overview of the costs incurred in each step are displayed in Tables 3 and 4, stratified by both patient and disease characteristics. Overall, the total cost of childbirth (SD) is €1894,12 (± 517,37). This cost is mainly driven by personnel costs (89,0%) of which midwives are the largest contributors (70,9%). Monitoring of the patient in the post-delivery phase costs €1149,70 and is the most expensive activity on the treatment path as it is highly personnel-intensive.

**Table 3:** Cost of childbirth for vaginal delivery broken down by patient characteristics (€ per patient)

	Average VD	Age		Gestation		Parity		Education	
		≤25 (*n* = 7)	> 25 (*n* = 13)	Normal (*n* = 11)	Long (*n* = 9)	Nulliparous (n = 7)	Multiparous (n = 13)	Low (*n* = 4)	High (n = 4)
**Pre-delivery**									
**Registration**									
Personnel	10.26	10.26	10.26	10.26	10.26	10.26	10.26	10.26	10.26
Infrastructure	0.14	0.14	0.14	0.14	0.14	0.14	0.14	0.14	0.14
Overhead	0.09	0.09	0.09	0.09	0.09	0.09	0.09	0.09	0.09
Registration costs	10.49	10.49	10.49	10.49	10.49	10.49	10.49	10.49	10.49
**Clinical visit**									
Personnel	37.63	42.33	35.10	35.14	40.69	48.77	31.64	29.58	40.28
Infrastructure	0.50	0.56	0.47	0.47	0.54	0.65	0.42	0.39	0.54
Overhead	0.30	0.34	0.28	0.28	0.33	0.39	0.25	0.24	0.32
Clinical visit costs	38.44	43.24	35.85	35.88	41.56	49.81	32.31	30.21	41.14
**Delivery**									
**First phase**									
Personnel	472.80	384.72	520.23	253.01	741.42	497.39	459.55	433.81	450.71
Infrastructure	6.29	5.12	6.93	3.37	9.87	6.62	6.12	5.78	6.00
Overhead	3.78	3.07	4.16	2.02	5.92	3.97	3.67	3.47	3.60
First phase costs	482.87	392.91	531.31	258.40	757.22	507.99	469.34	443.05	460.31
**Second phase**									
Personnel	27.66	34.13	24.18	29.40	25.54	49.10	16.12	17.75	41.13
Infrastructure	0.37	0.45	0.32	0.39	0.34	0.65	0.21	0.24	0.55
Overhead	0.22	0.27	0.19	0.23	0.20	0.39	0.13	0.14	0.33
Second phase costs	28.25	34.85	24.70	30.02	26.08	50.14	16.47	18.12	42.01
**Aftercare**									
Personnel	144.34	142.46	145.35	134.91	155.87	137.15	148.22	126.76	139.44
Infrastructure	1.92	1.90	1.94	1.80	2.08	1.83	1.97	1.69	1.86
Overhead	1.15	1.14	1.16	1.08	1.25	1.10	1.18	1.01	1.11
Aftercare costs	147.42	145.49	148.45	137.78	159.19	140.07	151.37	129.46	142.41
**Post-delivery**									
**Monitoring**									
Personnel	918.18	936.78	908.17	887.90	955.19	896.82	929.68	907.40	1206.33
Infrastructure	61.12	62.35	60.45	59.10	63.58	59.69	61.88	60.40	80.30
Overhead	36.67	37.41	36.27	35.46	38.15	35.82	37.13	36.24	48.18
Monitoring costs	1015.97	1036.54	1004.89	982.46	1056.91	992.33	1028.69	1004.03	1334.81
**Checkout**									
Personnel	6.8	6.8	6.8	6.8	6.8	6.8	6.8	6.8	6.8
Infrastructure	0.09	0.09	0.09	0.09	0.09	0.09	0.09	0.09	0.09
Overhead	0.06	0.06	0.06	0.06	0.06	0.06	0.06	0.06	0.06
Checkout costs	6.96	6.96	6.96	6.96	6.96	6.96	6.96	6.96	6.96
Medicines	28.59	25.13	30.45	19.99	39.09	25.13	30.45	32.99	21.99
Medical consumables	49.68	48.45	50.35	45.03	55.36	64.72	41.59	39.06	47.28
**Total cost** (mean)	1808.66	1744.06	1843.44	1527.03	2152.87	1847.64	1787.67	1714.38	2107.39
Standard deviation	± 490.96	± 571.22	± 463.42	± 350.29	± 420.42	± 492.04	± 509.15	± 263.60	± 406.99

**Table 4:** Cost of childbirth for caesarean section broken down by patient characteristics (€ per patient)

	Average CS	Age		Gestation		Parity		Education	
		≤25 (n = 2)	> 25 (n = 1)	Normal (n = 1)	Long (n = 2)	Nulliparous (n = 2)	Multiparous (n = 1)	Low (n = 1)	High (n = 1)
**Pre-delivery**									
**Registration**									
Personnel	10.26	10.26	10.26	10.26	10.26	10.26	10.26	10.26	10.26
Infrastructure	0.14	0.14	0.14	0.14	0.14	0.14	0.14	0.14	0.14
Overhead	0.09	0.09	0.09	0.09	0.09	0.09	0.09	0.09	0.09
Registration costs	10.49	10.49	10.49	10.49	10.49	10.49	10.49	10.49	10.49
**Clinical visit**									
Personnel	75.12	95.78	33.80	157.75	33.80	33.80	157.75	33.80	33.80
Infrastructure	1.00	1.28	0.45	2.10	0.45	0.45	2.10	0.45	0.45
Overhead	0.60	0.77	0.27	1.26	0.27	0.27	1.26	0.27	0.27
Clinical visit costs	76.72	97.82	34.52	161.11	34.52	34.52	161.11	34.52	34.52
**Delivery**									
**Baby Delivery**									
Personnel	12.39	12.96	11.27	13.52	11.83	11.83	13.52	11.27	12.39
Infrastructure	0.17	0.17	0.15	0.18	0.16	0.16	0.18	0.15	0.17
Overhead	0.10	0.10	0.09	0.11	0.09	0.09	0.11	0.09	0.10
Delivery costs	12.66	13.23	11.51	13.81	12.08	12.08	13.81	11.51	12.66
**Recovery Time**									
Personnel	101.41	101.41	101.41	67.61	118.31	118.31	67.61	101.41	135.21
Infrastructure	1.35	1.35	1.35	0.90	1.58	1.58	0.90	1.35	1.80
Overhead	0.81	0.81	0.81	0.54	0.95	0.95	0.54	0.81	1.08
Baby delivery costs	103.57	103.57	103.57	69.05	120.83	120.83	69.05	103.57	138.09
**Aftercare**									
Personnel	146.48	36.62	366.20	50.71	194.37	194.37	50.71	366.20	22.54
Infrastructure	1.95	0.49	4.88	0.68	2.59	2.59	0.68	4.88	0.30
Overhead	1.17	0.29	2.93	0.41	1.55	1.55	0.41	2.93	0.18
Aftercare costs	149.60	37.40	3740.00	51.79	198.51	198.51	51.79	3740.00	23.02
**Post-delivery**									
**Monitoring**									
Personnel	1791.66	1733.33	1908.32	1562.51	1906.23	1906.23	1562.51	1908.32	1904.15
Infrastructure	119.26	115.37	127.02	104.00	126.88	126.88	104.00	127.02	126.74
Overhead	71.55	69.22	76.21	62.40	76.13	76.13	62.40	76.21	76.05
Monitoring costs	1982.47	1917.93	2111.55	1728.91	2109.24	2109.24	1728.91	2111.55	2106.94
**Checkout**									
Personnel	6.8	6.8	6.8	6.8	6.8	6.8	6.8	6.8	6.8
Infrastructure	0.09	0.09	0.09	0.09	0.09	0.09	0.09	0.09	0.09
Overhead	0.06	0.06	0.06	0.06	0.06	0.06	0.06	0.06	0.06
Checkout costs	6.96	6.96	6.96	6.96	6.96	6.96	6.96	6.96	6.96
Medicines	29.32	21.99	43.98	43.98	21.99	21.99	43.98	43.98	0
Medical consumables	92.20	92.20	92.20	92.20	92.20	92.20	92.20	92.20	92.20
**Total cost** (mean)	2463.98	2301.58	2778.78	2178.29	2606.83	2606.83	2178.29	2788.78	2424.88
Standard deviation	± 307.12	± 174.36	NA	NA	± 257.32	± 257.32	NA	NA	NA

Note: The sample of caesarean section consists of only three patients. Consequently, the total cost when age > 25 years and high education is the same as it refers to one patient. Similarly, the cost when gestation long equals the cost when nulliparous, and the cost when gestation normal equals multiparous. Caution is advised when inferring conclusions

The cost of childbirth varies significantly between patients having a VD and a CS. The cost for a VD equals €1808,66 (± 490,96) relative to the cost for CS of €2463,98 (± 307,12). The higher personnel costs associated with the longer duration of the clinical visit (+ 33,3 min) and the monitoring after birth (+ 775,2 min) in CS are the main drivers explaining this cost difference. This difference is partly counterbalanced by the delivery phase which requires more time for a VD (+ 341,25 min). The cost of medicines is approximately equal for both types of delivery, while medical consumable costs are higher for CS.

Within the group of VD, there is substantial variability in cost of childbirth according to patient characteristics. First, the childbirth cost of older patients (€1843,44) is higher than the cost of younger patients (€1744,06) primarily due to the shortening and opening of the cervix (first phase) that takes longer among older patients (+ 120,3 min). Second, nulliparous women have a higher childbirth cost (€1847,64) than multiparous women (€1787,67) because of. a more prolonged first phase (+ 33,6 min) and second phase (+ 29,3 min) for nulliparous women. Third, cost is higher for women delivering after more than 40 weeks’ gestation (€2152,87) compared to gestation between 37 and 40 weeks (€1527,03). The major difference lies again in the longer first phase (+ 433,5 min) after more weeks of gestation. Lastly, having a higher education attainment leads to a higher childbirth cost (€2107,39) relative to lower education attainment (€1714,38), caused by an overall longer duration of the individual activities.

Within the group of women having a CS, there is a comparable variability in cost of childbirth according to age, parity and number gestation weeks at moment of childbirth. Nulliparous (€2606,83), older than 25 years (€2778,78) and birth after 40 gestation weeks (€2606,83) lead to higher childbirth cost relative to multiparous (€2178,29), younger (€2301,58) and birth before 40 gestation weeks (€2178,29) due to additional recovery time and aftercare. In contrast to VD, lower education attainment (€2788,78) is associated with a higher childbirth cost than higher education (€2424,88) as a result of an overall longer treatment path.

## Discussion

Health care organizations around the world are under increasing pressure to improve resource utilization and to become more cost effective. At the core of this problem is the lack of accurate cost information associated with specific medical interventions. Recently, time-driven activity-based costing is put forward as suitable, more precise costing method to provide detailed and dynamically cost insights in a complex environment such as hospitals [10, 18, 21]. The purpose of this study was the application of a time-driven activity-based method in order to estimate the cost of childbirth at a maternal department. Moreover, this study sought to extend prior literature by showing how the TDABC costing method can be used to outline how variation in childbirth cost is driven by specific patient and disease characteristics. Acting on the recommendations of Keel et al. [21], this study followed and reported the seven-step approach explicitly to deepen understanding of the costs incurred during the full cycle of care of a woman giving birth.

First, this study shows how time-driven activity-based costing starts from a documented process map, together with the inclusion of utilized resources and time estimates. Capacity cost rates were calculated for each resource and cost was estimated subsequently. We found that the vast majority of total cost of childbirth is driven by personnel costs. In particular, monitoring after birth was the activity along the pathway consuming most personnel resources and thus contributed heavily to overall cost.

Second, our time-driven activity-based analysis indicated that a substantial variability in costs of childbirth can be observed both between and within types of delivery. Type of delivery impacted the treatment path followed by patients and therefore resulted in different cost outcomes. In line with findings from prior literature, we found that overall cost of childbirth for women having a CS is higher than for a VD [17, 19, 23]. This cost difference could be primarily attributed to higher personnel costs associated with a longer clinical visit and the monitoring after birth, as well as higher use of medical consumables for CS. Additionally, the results of this study revealed several patient characteristics that drive variability in childbirth cost. Within the group of VD, substantial cost fluctuations could be identified according to age, parity, number of gestation weeks and education attainment. Women over twenty-five years were shown to need more time at the first phase of labour than younger women, leading to a higher cost. These results could be explained by an increased weakness of muscles with aging, resulting in less effective and more extended contractions, which are consistent with prior studies [6, 17, 19, 24]. Similarly, women giving birth to their first child and women delivering after more gestation weeks were also associated with a longer first phase. In contrast to multiparous women, nulliparous women do not easily have a spontaneous rupture of the membranes, clarifying a more prolonged first phase of labour [14]. A higher birth weight associated with a slower labour explains the longer first phase for women delivering after more gestational weeks [14]. Finally, a higher education attainment was found to lead to higher cost of birth because of overall prolonged time estimations over the complete clinical pathway, which also confirms prior documented research [6]. Health awareness can lead to more care-seeking behaviour for maternal health services, explaining more clinical time needed for higher educated women [20]. Within CS, similar cost variations could be identified according to age, parity and number of gestations weeks. Supporting prior literature, we found initial evidence that costs of CS increase along with a woman’s age, which could be explained by older women being more susceptible for complications and difficulties during CS, resulting in more clinical care and prolonged hospitalization [6, 17, 19, 25]. Furthermore, this study suggested that women delivering their first child, and women giving birth after more than 40 gestation weeks had a higher cost. In contrast to VD, where higher cost was driven by a longer first phase, our findings however indicated that these women required prolonged aftercare and monitoring after birth. This could also be caused by an increased complexity and risk of major issues for women delivering after more gestation weeks and nulliparous status [13–15]. Lastly, our results indicated that women having a lower education attainment have a higher cost of childbirth, primarily due to longer aftercare. The impact of this characteristic on costs differs for VD and the findings are moreover inconsistent with prior literature, where it is found that a higher education attainment is associated with increased costs for CS [6, 20]. However, it is important to note that our outcome is based on only two observations and thus should not be generalized.

Our study provides new perspectives on the estimation of cost of childbirth in a hospital, and on the determinants driving cost variability. First*,* while a number of studies have already identified that age, parity and education attainment can drive variability in overall cost of childbirth, our study differs from existing studies in the use of the time-driven activity-based costing approach to analyse the impact of several patient and disease characteristics on cost of childbirth [6, 17–20, 26]. In addition to providing more accurate cost estimates than traditional methods, this technique allows to exactly identify which activities and resources are most heavily impacted by these characteristics [26]. Despite a growing popularity in other medical disciplines, we did not find any study that uses this technique to estimate and analyse cost variability in childbirth with one exception [9, 10, 18, 21–23]. One study implemented in their paper a time-driven activity-based approach to identify cost determinants of a CS [18]. However, this study was performed in Rwanda, where health systems and quality of care are very different in comparison to more developed countries, and the study did not include VD in its scope. Second, our study is also relevant to many applications in other hospital treatments. By explicitly outlining the seven step-by-step approach of Kaplan and Porter, we could guide future initiatives aiming to set up similar short-series using time-driven activity based costing [9, 22].

However, there is also an important limitation to this study. The relatively small size of the sample limits the generalizability of the findings regarding cost estimations. In particular for CS, not many conclusions on the quantitative impact of patient characteristics on cost of birth can be drawn due to the limited number of observations. Nevertheless, this study shows that time-driven activity is a suitable costing method to estimate cost of childbirth and to account for cost variabilities. Future research could elaborate on this study by extending the sample frame. Such an extension could moreover allow researchers to apply statistical analyses to examine the impact of multiple characteristics on cost differences, thereby further investigating the proposed relationships.

## Conclusions

Accurate cost information on the provided care is crucial for health care providers and managers to cope with increasing cost pressures. This study adds to the growing research body on the use of time-driven activity-based costing in health care organizations, showing how TDABC can be used as a valuable tool to measure and better understand the total cost and variabilities of childbirth over the full cycle of care. Moreover, we provide initial insights into drivers of this variability. In particular, type of delivery was found to impact the decisions on the clinical pathway leading to different activities, while patient characteristics mainly drive the duration of activities in the delivery phase. Hence, the findings of our study have important implications for practice. This research demonstrates how TDABC is advantageous for operational improvements at the maternity department by offering transparency into non-value adding activities. Since process maps and time equations expose the cost of activities in detail, health care providers and managers can identify opportunities for the reduction of resource waste, waiting times and redundant steps concerning the process of childbirth. Simultaneously, TDABC can also inform policymakers and hospital managers on reimbursement schemes and profitability.

## Supplementary Information

**Additional file 1.** English survey.

**Additional file 2.** Dutch survey (original version).

**Additional file 3.** Checklist STROBE guidelines.

## Data Availability

The datasets used and/or analysed during the current study are available from the corresponding author on reasonable request.

## Abbreviations

TDABC: Time-driven activity-based costing
VD: Vaginal delivery
CS: Caesarean section
COPD: Chronic obstructive pulmonary disease
CDVC: Care delivery value chain
CCR: Capacity cost rate
