# Correction to: A time-driven activity-based costing approach for identifying variability in costs of childbirth between and within types of delivery

**DOI:** 10.1186/s12884-021-04253-y

**Published:** 2021-11-25

**Authors:** Kathia Dubron, Mathilde Verschaeve, Filip Roodhooft

**Affiliations:** 1 KU Leuven, University Hospital Leuven, Kapucijnenvoer 33, 3000 Leuven, Belgium; 2 KU Leuven, Faculty of Economics and Business, Research Centre Accountancy, Leuven, Belgium; 3 Vlerick Business School, Accounting and Finance, Ghent, Belgium

**Correction to: BMC Pregnancy Childbirth 21, 705 (2021)**

**https://doi.org/10.1186/s12884-021-04134-4**

Following publication of the original article [1], the authors reported an error in the response letter/peer review reports to the editors. The name of the hospital should be removed from the authors’ response letter.

The original article [1] has been updated.
