# Supplementary material for: A time-driven activity-based costing approach for identifying variability in costs of childbirth between and within types of delivery
Source: BMC Pregnancy Childbirth. 2021 Oct 20;21:705. doi: 10.1186/s12884-021-04134-4 (PMC8527632; doi:10.1186/s12884-021-04134-4)
Supplement: Supplementary file 1 — Additional file 1. English survey. [file 12884_2021_4134_MOESM1_ESM.docx]

*(Anonymous) Examination number*: **…**

**PART 1**

**1.**

**TIME INTAKE = ……………**

**2.**

**DURATION OF CLINICAL EXAMINATION ON ADMISSION = ……………**

**3.**

**DURATION OF THE FIRST PHASE = ……………**

| Physical examination | Blood pressure  Measurement | CTG Actions | Vaginal touch | Echo Abdomen | Transvaginal  Echo | Blood lab |
| --- | --- | --- | --- | --- | --- | --- |
| ***How many times?*** |  |  |  |  |  |  |

**4.**

**DURATION (TIME) OF DELIVERY = ……………**

*(please circle what applies)*

| **VAGINAL** | **CAESAREAN SECTION** |
| --- | --- |
| Morphine pump / epidural / paracetamol drip / other | General anaesthesia / lumbar puncture |
| Induction / spontaneous | Planned / acute |
| Episiotomy YES / NO | Complications YES / NO |
| Forceps YES / NO |  |
| Vacuum pump YES / NO |  |
| Complications YES / NO |  |
| Bath-birth YES / NO |  |

**5.**

| *(on obstetrics)*  **DURATION OF AFTERCARE = …….** | **RECOVERY TIME = …….** |
| --- | --- |
| *(e.g., suturing, washing,* *breastfeeding)* | *(waking up)* |
|  | *(on maternity)*  **DURATION OF AFTERCARE = …….** |

*(e.g., washing,* *breastfeeding)*

**6.**

**AFTER CHILDBIRTH**

**Time and day of discharge = ……………**

**Number of days of admission = ……………**

*(Anonymous) Examination number*: **…**

**PART 2: PATIENT BACKGROUND**

1. **AGE OF PATIENT: …………. years**
2. **EDUCATION LEVEL (please circle what applies)**

| PRIMARY SCHOOL | HIGH SCHOOL | COLLEGE DEGREE | UNIVERSITY |
| --- | --- | --- | --- |

1. **ANAMNESIS** *(please circle what applies)*

| **Are there any heart diseases?** | **YES** | **NO** |
| --- | --- | --- |
| *If yes, which one?*  - Hypertension  - Pre-eclampsia | *MODERATE*  YES | *SEVERE*  *NO* |
| **Does the patient have diabetes?** | **YES** | **NO** |
| *- If yes, is it gestational diabetes?* | *YES* | *NO* |
| **Are there any lung diseases?** | **YES** | **NO** |
| *If yes, which one?*  - COPD  - Asthma | *MODERATE MODERATE* | *SEVERE SEVERE* |
| **Intoxications present?** | **YES** | **NO** |
| *If yes, which one?*  - Smoking  - Alcohol | *YES*  **YES** | NO  NO |
| **30<BMI<40** | **YES** | **NO** |

1. **PARITY** *(please circle what applies)*

| NULLIPAROUS | MULTIPAROUS |
| --- | --- |
|  | Medical History: CAESAREAN SECTION YES/ NO |
|  | Medical History: VAGINAL YES/ NO |

1. **LANGUAGE LEVEL** *(please circle what applies)*

| DUTCH | FRENCH | OTHER |
| --- | --- | --- |

1. **NUMBER OF GESTATION WEEKS** *(please circle what applies)*

| <37 weeks | 37-40 weeks | >40 weeks |
| --- | --- | --- |

1. **POVERTY NETWORK** *(please circle what applies)*

| JA | NEEN |
| --- | --- |

1. **ROOM** *(please circle what applies)*

| PRIVATE ROOM | DOUBLE ROOM | LUXERY ROOM |
| --- | --- | --- |
