# Supplementary material for: A time-driven activity-based costing approach for identifying variability in costs of childbirth between and within types of delivery
Source: BMC Pregnancy Childbirth. 2021 Oct 20;21:705. doi: 10.1186/s12884-021-04134-4 (PMC8527632; doi:10.1186/s12884-021-04134-4)
Supplement: Supplementary file 2 — Additional file 2. Dutch survey (original version). [file 12884_2021_4134_MOESM2_ESM.docx]

*(Anoniem) Onderzoeksnummer* : **…**

**DEEL 1**

**1.**

**TIJDSTIP OPNAME = ……………**

**2.**

**TIJDSDUUR KLINISCH ONDERZOEK BIJ OPNAME = ……………**

**3.**

**TIJDSDUUR ONTSLUITINGSFASE = ……………**

| Lichamelijk Onderzoek | Bloeddruk  Meting | CTG Handeling | Vaginaal toucher | Echo Buik | Transvaginale  Echo | Labo |
| --- | --- | --- | --- | --- | --- | --- |
| ***Hoe vaak?*** |  |  |  |  |  |  |

**4.**

**TIJDSDUUR BEVALLING = ……………**

*(omcirkel wat plaatsgevonden heeft)*

| **VAGINAAL** | **KEIZERSNEDE** |
| --- | --- |
| Morfinepomp / ruggenprik / paracetamol infuus / anders | Algemene anesthesie / ruggenprik |
| Inleiding / spontaan | Gepland / acuut |
| Episiotomie JA / NEEN | Complicaties JA / NEEN |
| Verlostang JA / NEEN |  |
| Vacuümpomp JA / NEEN |  |
| Complicaties JA / NEEN |  |
| Bad-bevalling JA / NEEN |  |

**5.**

| *(op verloskunde)*  **TIJDSDUUR NAZORG = …….......** | **TIJDSDUUR RECOVERY = …………….** |
| --- | --- |
| *(vb; hechting, wassen, aanleggen..)* | *(wakker worden)* |
|  | *(op materniteit)*  **TIJDSDUUR NAZORG = …….......** |

*(vb: wassen, aanleggen..)*

**6.**

**NA DE BEVALLING**

**Tijdstip en dag ontslag = ……………**

**Aantal dagen van opname = ……………**

*(Anoniem) Onderzoeksnummer* : **…**

**DEEL 2: ACHTERGROND PATIENTE**

1. **LEEFTIJD PATIENTE: ………….jaar**
2. **OPLEIDINGSNIVEAU** *(omcirkel wat past)*

| BASISSCHOOL | MIDDELBAAR | HOGESCHOOL | UNIVERSITEIT |
| --- | --- | --- | --- |

1. **ANAMNESE** *(omcirkel wat past)­­­­*

| **Zijn er hartaandoeningen?** | **JA** | **NEEN** |
| --- | --- | --- |
| *Zo ja welke?*  - Hypertensie  - Pre-eclampsie | *MATIG*  *JA* | *ERSTIG*  *NEEN* |
| **Heeft de patiënte diabetes?** | **JA** | **NEEN** |
| *- Zo ja, zwangerschapsdiabetes?* | *JA* | *NEEN* |
| **Zijn er longaandoeningen?** | **JA** | **NEEN** |
| *Zo ja welke?*  - COPD  - Astma | *MATIG MATIG* | *ERNSTIG ERNSTIG* |
| **Intoxicaties aanwezig** | **JA** | **NEEN** |
| *Zo ja welke?*  - Roken  - Alcohol | *JA*  *JA* | *NEEN*  *NEEN* |
| **30<BMI<40** | **JA** | **NEEN** |

1. **PARITEIT** *(omcirkel wat past)*

| PRIMIPARA | MULTIPARA |
| --- | --- |
|  | Voorgeschiedenis: KEIZERSNEDE JA / NEE |
|  | Voorgeschiedenis: VAGINAAL JA / NEE |

1. **TAALNIVEAU** *(omcirkel wat past)*

| NEDERLANDS | FRANS | ANDERS |
| --- | --- | --- |

1. **AANTAL WEKEN ZWANGER** *(omcirkel wat past)*

| <37 weken | 37-40 Weken | >40 weken |
| --- | --- | --- |

1. **KANSARM** *(omcirkel wat past)*

| JA | NEEN |
| --- | --- |

1. **KAMER** *(omcirkel wat past)*

| EENPERSOONS KAMER | TWEEPERSOONS KAMER | LUXE |
| --- | --- | --- |
